# Factors Associated with Food Insecurity among Pregnant Women and Caregivers of Children Aged 0–6 Years: A Scoping Review

**DOI:** 10.3390/nu14122407

**Published:** 2022-06-09

**Authors:** Amber Bastian, Courtney Parks, Amy Yaroch, Fiona H. McKay, Katie Stern, Paige van der Pligt, Sarah A. McNaughton, Rebecca Lindberg

**Affiliations:** 1 Institute for Physical Activity and Nutrition (IPAN), School of Exercise and Nutrition Sciences, Deakin University, Locked Bag 2000, Geelong, VIC 3220, Australia; a.bastian@deakin.edu.au (A.B.); p.vanderpligt@deakin.edu.au (P.v.d.P.); sarah.mcnaughton@deakin.edu.au (S.A.M.); 2 Gretchen Swanston Centre for Nutrition, 8401 W Dodge Rd, Omaha, NE 68114, USA; cparks@centerfornutrition.org (C.P.); ayaroch@centerfornutrition.org (A.Y.); k.stern@centerfornutrition.org (K.S.); 3 Institute for Health Transformation, School of Health and Social Development, Deakin University, Locked Bag 2000, Geelong, VIC 3220, Australia; fiona.mckay@deakin.edu.au

**Keywords:** food security, food insecurity, pregnancy, young children

## Abstract

With a global focus on improving maternal and child nutrition through the 2030 Sustainable Development Goals, it is important to understand food insecurity in pregnant women and families with young children, as food insecurity at these life stages can have ongoing negative health consequences. However, factors that influence food insecurity among this population group are not well understood. This scoping review investigates the factors that influence food insecurity among pregnant women and households with young children aged 0–6 years living in high-income countries. A scoping literature review was conducted using four electronic databases. The search combined terms relevant to: food security, determinants, pregnancy and family and high-income countries. Only full text and English language articles were included. The search identified 657 titles and abstracts; 29 articles were included in the review. A majority (70%) of the studies were conducted in the United States and were mostly either cross-sectional or secondary data analysis of existing population data. Factors associated with food insecurity were identified and grouped into 13 constructs. These included social, economic and health risk factors, food access and utilization factors and health and dietary outcomes. This scoping review identifies the factors associated with food insecurity among pregnant women and families with young children that could be used to better measure and understand food insecurity, which could assist in developing program and policy responses. This review also highlights the lack of literature from high-income countries outside the US.

## 1. Introduction

Despite increased access to resources and economic advantages, approximately 10% of people living in high-income countries (HIC) experience food insecurity, with factors relating to food insecurity varying by level of development and Gross Domestic Product per capita [1]. After controlling for economic factors, some household characteristics can increase the risk of experiencing food insecurity. Households are more likely to experience food insecurity if they include single women with children [2], ethnic minority populations [2], households with grandchildren present [3], households with a disabled parent or child [4], households with someone who is currently or previously incarcerated [5] and households with an adult smoker [6].

Food insecurity can affect all stages of the lifespan; however, women are at high risk of becoming food insecure due to entrenched societal power inequality and a range of socio-economic conditions such as domestic violence, poor employment, and education [7,8]. Households with children are at increased risk of food insecurity, as such, mothers and in particular single mothers experience a higher rate of food insecurity compared with women without children [7]. Living in a food insecure household during pregnancy may increase risk of greater gestational weight gain, disordered eating, chronic disease and pregnancy complications [9] while the impact of food insecurity on young children is particularly concerning given they are at a key stage of growth and development which can influence health during adolescence and even adulthood [10]. Infants residing in food insecure households are more likely to have poor health, be nutrient deficient and be hospitalized [11,12] with poor health and developmental challenges, including cognitive, linguistic, social, and emotional challenges being more common among food insecure children [13]. While there are clear negative health outcomes for food insecure households with pregnant women and young children, the coping mechanisms employed by these households are not well understood. Studies have reported that mothers experiencing food insecurity are likely to engage in coping strategies such as delaying payments of bills, giving up services, selling or pawning possessions and diluting infant formula [14,15].

Globally, there is a commitment to improve maternal and child nutrition across countries through the 2030 Sustainable Development Goals. Understanding factors that threaten pregnant women and caregivers of young children’s food supply and nutritional adequacy is fundamental to supporting both the short- and long-term health of women and children. It is important to understand food insecurity in this population as the problem is widespread and impacts women and children in HIC as well as low–medium income countries. Collectively, in HIC, evidence which best summarises factors associated with food insecurity is lacking. Furthermore, factors associated with food insecurity in HIC may be different from those associated with food insecurity in low–medium income countries. Identifying such factors is essential to understand and inform how and when strategies may be implemented which specifically target food insecurity in this population group.

Existing literature reviews have summarized the factors that influence food insecurity in the general population, as well as outcomes potentiated by the experience of food insecurity. Many reviews have focused specifically on the United States (US) and have examined the factors influencing food insecurity, including food distribution, coping mechanisms (such as trade-offs between buying food and other expenses), the intersection of food insecurity and obesity [16,17,18] and chronic disease and health outcomes [10,19]. Literature reviews have focused on the experience of food insecurity among households with children [20] but have not been specific to pregnant women and households with young children under the age of 6. Reviews that explore the food security situation across more than one country include rates of food insecurity in post-secondary education campuses [21], food insecurity measurement tools utilized in HIC [22] and the impact of local environmental characteristics on food insecurity, including social capital, crime, and access to food stores [23]. Despite these reviews spanning geographies and topics, the factors associated with food insecurity among pregnant women and caregivers of young children in HICs are not well understood. As scoping reviews are useful tools to scope a body of literature, identify knowledge gaps and key characteristics or factors related to a concept [24], this review seeks to uncover the factors associated with food insecurity in this population. The aim of this scoping review is to identify the factors that influence food insecurity among pregnant women and caregivers of young children living in HIC.

## 2. Materials and Methods

This scoping review was conducted to investigate the factors that influence food insecurity among pregnant women and households with young children (aged 0–6 years) in HICs. A comprehensive systematic search informed by Peters et al. [25] was conducted in four databases: Medline complete, Embase, Global Health, and CINAHL. These databases were chosen to provide coverage of public health nutrition and nursing and allied health literature in HIC including the US and Europe. Search terms (Appendix A, Table A1) were relevant to: food insecurity, determinants, pregnancy and family, and HIC as defined by the Human Development Index as having a higher life expectancy, education and per capita income indicators [26]. The search strategy involved combining the search terms and all terms were searched in title and/or abstract. To ensure inclusion of recent literature the search was limited to the last 15 years (date range: 1 January 2005 to 1 April 2020) and articles published in English. The search was conducted on 21 April 2020.

References were imported into Covidence [27] to manage the screening process. To determine inclusion, two phases of screening were conducted. Firstly, two reviewers independently screened the titles and abstracts for inclusion. This was followed by full-text screening to remove irrelevant articles based on the exclusion criteria. Any conflicts were resolved by a third reviewer. Table 1 outlines the inclusion and exclusion criteria, developed using the PICO/PICo frameworks, in terms of population (pregnant women and care givers with children aged 0–6), outcome (food security) and context (HIC) [28]. Articles were excluded if for example they included the wrong population group [29], were systematic or systematic-like reviews [30], or did not explicitly mention food insecurity among the target population group [31]. Full-text articles were read, and relevant information was extracted using predetermined categories including country of study, setting/population, study design, food insecurity measurement tool used and outcomes. Consistent with the approach taken in scoping reviews, articles were not accessed for quality, rather the various factors related to food security were extracted [24]. The range of factors were discussed by the research team and grouped into a series of overarching constructs. Results were summarised and organised into these key constructs that influence food security and insecurity. The research team reviewed and discussed the extracted data and results to ensure consistency across reviewers.

## 3. Results

**Figure 1:**
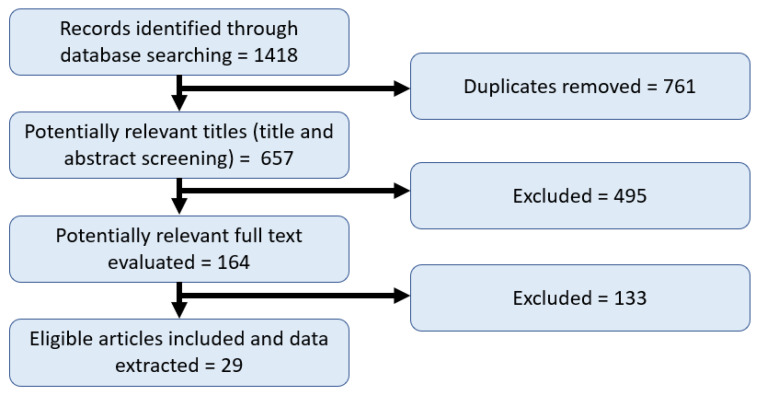
Literature review study selection flowchart.

The initial search resulted in 1418 articles, of which 716 were duplicates. The title and abstract of the remaining 657 articles were read; 495 were excluded as they did not meet the inclusion criteria. The full text of the remaining 164 studies were read with a further 133 excluded as they did not meet inclusion criteria. Reasons for exclusion included wrong population group, study design or outcomes as outlined in Table 1. A total of 29 relevant studies were included for analysis (Figure 1). Most studies were conducted in the US (*n* = 20), with the remaining from Canada (*n* = 3), Malaysia (*n* = 2), Argentina (*n* = 2), Mexico and US (*n* = 1), and New Zealand (*n* = 1). Most studies employed a cross-sectional design (*n* = 22) and just over half were secondary data analysis of existing population data sets (*n* = 16), while three studies employed qualitative methodology. A majority of studies (*n* = 20) used a version of the United States Department of Agriculture (USDA) Food Security Module to measure food security status, with the Radimer/Cornell hunger scale being the second most commonly used measure (*n* = 4). One of the studies included pregnant women only, none of the studies focused on single caregivers specifically. Details of the 29 included studies can be found in Appendix B, Table A2.

Analysis revealed 13 constructs relating to factors associated with food security. Table 2 lists these constructs and identifies the factors as positively associated with food security (+) or inversely associated with food security (−).

The main findings of this scoping review are organised into 13 constructs as outlined in Table 2. Sociodemographic factors were included in 17 of the 29 articles. These factors include income and employment [32,33,34,35,36,37,38,39,40,41,42,43,44], education [35,37,40,41,45,46,47], ethnicity [33,36,37,39,46,48] and family composition [41,43,46,47] as the main factors associated with food insecurity for pregnant women and caregivers of young children.

Coping strategies were mentioned in eight papers [32,34,35,36,43,45,49,50]. Coping strategies include utilising social support and making trade-offs about what to eat, who eats and how often meals are eaten. These behaviours are associated with an increased likelihood of food insecurity but also as a mechanism to protect against food insecurity. Maternal depression and mental health were also established in eight papers [36,42,46,47,48,49,51,52]. Maternal depression was associated with increased likelihood of food insecurity [46,53] but also, as a health outcome associated with the stress and anxiety of food insecurity [42,48]. Parenting stress included lack of time and social support and feelings of isolation, all of which may be barriers to food security for some families [49]. In addition, Patel and Surkin (2016) found children born to parents who jointly reported unplanned childbearing were more likely to be exposed to household food insecurity in their first two years of life, independent of other factors.

Residence stability and crowding were included in seven papers associated with increased rates of food insecurity [34,39,40,43,45,46,54]. Parent acculturation, which includes length of time spent living in the country of study and immigrant status, were a focus of six studies [35,46,55,56,57,58]. Interestingly, one paper found length of time living in the country of study was not associated with household food insecurity [55] while the other five papers did find an association. Hadley and Sellen (2006) highlight how a more recent arrival in the US is associated with difficulty shopping and preparing food in a foreign environment, both of which can increase food insecurity. In addition, immigrant status is linked to employment options and access to welfare benefits and food assistance programs, thus impacting food security. Six papers described how participation in government food assistance programs and/or charitable feeding program use is associated with food insecurity [35,36,48,50,59,60], however, only two found this to be protective against food insecurity [36,59]. Six papers mentioned diet quality, three highlighted how food insecurity was associated with reduced intakes of high-cost nutrient-rich foods and increased intake of low-cost nutrient-poor foods for both caregivers [55] and young children [38,39], two report an association between food insecurity and poor iron status in pregnant women [37] and young children [40] and one paper reports an association between food insecurity and reduced food variety [35]. Having a member of the household who was a smoker [37,39,45,47], who was a frequent user of health care [33] or who did not have health care coverage [37] placed additional financial strain on families and were found to negatively impact food security status as households prioritise spending money on other necessities over food [49,50]. Local neighbourhood infrastructure and characteristics [32], along with general access to food stores, and healthy affordable food stores specifically [32], emerged as other factors associated with food security from the literature.

## 4. Discussion

To our knowledge this is the first review that identifies a broad set of factors associated with food insecurity among pregnant women and caregivers of young children and group these findings into clear constructs. A total of 13 constructs relating to factors associated with food insecurity arose from the literature.

The literature included in this review covered a range of known risk factors for food insecurity including economic, social, and health. Economic factors including income and employment predict the financial resources of a household to purchase food and was the most frequently mentioned construct, with 13 papers highlighting a link between income-poverty and food insecurity. This is consistent with data from national surveys in the US which have routinely shown household income to be the strongest predictor of risk of food insecurity [61]. In other HIC, such as Australia, the average weekly full-time wage for women is 15.3% lower than for men, with women more likely to be employed part-time [62], thus reducing their earning capacity and placing them at risk of poverty. Lower income means a reprioritization of funds, food is replaced by accommodation costs, utility bills and other expenses [14]. Policy responses for food security need to address the underlying issue of inadequate income and available money to spend on food for women living in HICs.

Known social risk factors contributing to food insecurity include ethnicity, education level, marital status, family size and acculturation. These were all identified as common factors associated with food insecurity in households with pregnant women and/or young children and highlight the complexity in delivering relief strategies which might be useful in reducing food insecurity in this population group across HICs. For example, immigrating to a new country and the length of time spent living in that country not only impacts income and employment opportunities, but also knowledge of how to access welfare services and difficulties with shopping and food preparation in a foreign environment. Immigrant pregnant women/caregivers may need additional support to overcome barriers to nutritional adequacy and access to culturally appropriate foods. Social factors that are likely to be unique to this population group uncovered in this review include maternal depression, parenting stress, lack of time and social support, and feelings of isolation. Unplanned childbearing and its association to food insecurity was also a finding unique to this population group. Strategies to better support caregivers of young children and provide opportunities for them to socially connect could help alleviate some of these factors.

Poorer dietary and nutritional intakes are associated with diet-related chronic conditions such as diabetes and obesity [18], and elevated poor mental health including depression and anxiety [63]. Reduced intakes of high-cost nutrient-rich foods and increased intake of low-cost nutrient-poor foods have been observed for both caregivers and children [38,39,55] along with poor iron status in pregnant women and children [37,40]. Considering the fundamental and crucial role of adequate nutrition in supporting optimal pregnancy outcomes as well as optimal growth and development of children during the early years of life, it is essential that food insecurity is a key priority of public health programs supporting maternal and child health.

Studies among children suggest that they can recognise and experience the stress of food insecurity and that childhood experiences of hunger may be associated with poor physical and mental health outcomes into late adolescence [64]. This is consistent with the theory that the lived experience of food insecurity during critical periods of child development results in poor health outcomes later in life [10]. This highlights the importance of exploring factors associated with food insecurity during pregnancy and for families with young children, given that many adverse health, growth and development outcomes in early childhood development are well-known to track beyond childhood and into adverse health outcomes later in life [65].

The lack of literature on lived experience of household food insecurity in pregnant women and families with young children was evident in this scoping review. Only three of the 29 studies used a qualitative methodology to describe and understand the lived experience. Qualitative research methods enable researchers to delve into questions of meaning, examine institutional and social practices and processes, identify barriers and facilitators to change and discover the reasons for the success or failure of interventions [66]. Further qualitative studies on the lived experience of household food insecurity in pregnant women and families with young children could aid important understanding of the many complex factors associated with and resulting from food insecurity and offer insight into potential food security programs, policies and economic or social attributes required to improve food security for this population.

Just over half of the studies included in this review were based on secondary analysis of existing national data sets as opposed to primary research. Furthermore, the national data sets used for secondary analysis were used in multiple papers and are dated. For example, the Children’s Healthwatch (1998–2005) was used as the data set in four of the papers and these data are now over fifteen years old which raises questions around the applicability of the findings to the current context. A majority of the studies (70%) were conducted in the US with only five of the other 62 HIC represented in this review (Canada, Malaysia, Argentina, Mexico, and New Zealand). This is a research gap that needs to be urgently filled to increase understanding of factors contributing to food insecurity across families in other countries, contexts and among diverse cultural groups if the issue is to be tackled globally.

While there are a number of strengths to this review, including the identification of the 13 constructs, there are several limitations. One limitation is the majority of the studies included were conducted in the US. This may be related to the English inclusion criteria. While many countries use English, there is some research that may have been missed. One impact of this is that while all HIC were included in the search given that the majority of the research is from the US may mean the results are skewed to the US context. Future research could explore how the identified constructs changed if only US literature was examined versus including literature from other countries. Given the vast experiences between the HIC included in this search, it would be prudent for researchers to draw in research in other languages and for researchers in HIC outside of the US to continue to explore issues related to food insecurity among this population, as this will provide a fuller picture of the current experience. Furthermore, as scoping reviews do not attempt to appraise the quality of evidence, the constructs found within the literature should be interpreted with caution. Despite these limitations, this review identified a range of factors that influence food insecurity among pregnant women and households with young children that could be useful in future measurement, screening and strategies, and could be translated across a range of geographical contexts.

## 5. Conclusions

From this scoping review we have found a broad range of factors that have been grouped into 13 constructs associated with food insecurity that could be useful in measuring and understanding food insecurity in pregnant women and families with young children. Currently, the commonly used monitoring frameworks in HIC, may be too narrow. Capturing known economic, health and social risk factors such as recent job loss, maternal mental health and depression and family composition could be useful in screening families with young children and pregnant women at risk of food insecurity and planning and delivering appropriate policies and programs.

## Figures and Tables

**Table 1:** Inclusion and exclusion criteria used for abstract and full-text screening.

Inclusion Criteria	Exclusion Criteria
Original research articles Published in peer-reviewed journals Published between 1 January 2005 and 1 April 2020 Published in the English language Full text available Qualitative or quantitative studies (including descriptive studies, interventions/trials/RCTs including quasi-experimental and pre-post evaluations, prospective cohort studies, nested case-control studies) Studies in humans, families or households incorporating caregivers of young children and/or households with pregnant women. * If ages of children not specified in title and abstracts, they were included for full-text review. Studies conducted in populations in developed HIC (defined by the Human Development Index) Studies conducted in community dwelling or free-living populations Includes measures of any correlates or determinants of food security and includes one or more of the outcomes listed below: - Food Security (measured in anyway) - Other aspects of Food Security as defined by the Food and Agriculture Organization (FAO) (affordability, access, utilization, stability)	Conference proceedings and conference abstracts Not published in a peer-reviewed journal Not published between 1 January 2005 and 1 April 2020 Not published in English language Full text not available Opinion articles, systematic reviews, meta-analyses, narrative reviews, scoping reviews, study protocols, commentaries or case studies Studies of animals, families with children greater than 6 years (school aged children/adolescents, youth), adults only, women who were not pregnant. * If ages of children not specified in full text, they were excluded. If findings not presented for children 0–6 years, they were excluded. Studies not conducted in populations in developed HIC (defined by the Human Development Index) Studies not conducted in community dwelling or free-living populations Main outcomes were not food security (measured in anyway) or other aspects of food security not defined by the FAO

**Table 2:** Summary of constructs associated with food security arising from the literature.

Food Security Construct	No. of Papers	Details of Factors Associated with Food Security
Income and employment	13	Low income ^-^, job loss ^-^ and payment schedules ^-^, income support ^-^, caregiver unemployment ^-^, poverty ^-^, not receiving welfare ^-^, low social economic status ^-^ (combined score of education, occupation and household income), living below poverty line ^-^, mothers as housewives ^-^
Coping strategies	8	Stretching food^+ -^, going without^+ -^, caregivers/children skip meals or cut back on variety of foods consumed^+ -^, going to bed hungry^+ -^, other household members going hungry^+ -^, reducing the number of meals each day^+ -^, cooking whatever is available^+ -^, buying cheaper food^+ -^, shopping at value stores^+ -^, using coupons^+ -^, going to many locations to find best price^+ -^, reducing money spent on children’s education^+ -^, borrowing money^+ -^, social support and social cohesion^+^, contact with extended family and participation in food sharing networks^+ -^, consumption of meals outside the home (at other people’s homes)^+ -^, turning to family and friends^+ -^, receiving food from others^+ -^
Maternal depression/mental health	8	Maternal depression and poor health status ^-^, parenting stress ^-^, lack of time ^-^, lack of social support ^-^, feelings of isolation ^-^, unwanted child-bearing ^-^
Residence stability and crowding	7	Housing and energy insecurity ^-^, experience greater number of moves ^-^, receiving housing subsidy ^-^, do not own any land ^-^, household crowding ^-^, living in households with five or more members ^-^, crowding in homes in need of major repair ^-^
Education	7	Caregivers/mothers with lower levels of education ^-^
Parent acculturation	6	Immigrant status ^-^, length of time in country^+ -^, difficulty with shopping and food preparation in a foreign environment ^-^
Ethnicity	6	Ethnicity^+ -^, race^+ -^, ethnic minority ^-^
Participation in food assistance programs	6	Participation in SNAP^+ -^, participation in WIC ^+ -^, food stamp usage ^-^, reliance on school meals ^-^
Diet Quality	6	Reduced consumption of high cost and micronutrient rich foods ^-^, increased consumption of low-cost traditional staple foods ^-^, iron status ^-^, food variety ^-^
Family composition	4	Caregivers’ marital status (single/widowed/separated/divorced) ^-^, larger household size, larger number of children ^-^
Smoking	4	Living in a house with a smoker ^-^, maternal smoking ^-^, mother smoking during pregnancy ^-^
Food access and availability	4	Economic constraints ^-^ and food pricing (including cost of fruit and vegetables) ^-^, choosing between food and other necessities (including medicine and bills) ^-^, lack of access to healthy food ^-^ or food stores in general ^-^
Health care	2	Health care usage ^-^, lack of health insurance coverage ^-^
Other	2	Lack of urban infrastructure ^-^ and exposure to environmental contaminants ^-^

+ Positively associated with food security; - Inversely associated with food security.

## Data Availability

Not applicable.

## Appendix A

**Table A1:** Search terms.

Food Security	Determinants	Pregnancy and Families with Children Aged 0–6 Years	High-Income Countries
“food securit *” OR “food insecurit *” OR “hunger” OR “food pant *” OR “food bank *” OR “emergenc * food” OR “food access” OR “food availab *”	“factor” OR “factors” OR “etiolog *” OR “causl *” OR “causes” OR “determinant *” OR “ethnolog *” OR “component *” OR “dimension *” OR “influence *” OR “effect *” OR “association *” OR “characteristic *”	“child *” OR “infan *” OR “toddler *” OR “pre-school *” OR “preschool *” OR “kindergarten” OR “parent *” OR “mother *” OR “father *” OR “family” OR “families” OR “pregnan *” OR “gestation *” OR “maternal health” OR “prenatal” OR “pre-natal” OR “postnatal” OR “post-natal”	“andorra” OR “argentina” OR “australia” OR “austria” OR “bahamas” OR “bahrain” OR “barbados” OR “belarus” OR “belgium” OR “brunei darussalam” OR “bulgaria” OR “canada” OR “chile” OR “croatia” OR “cyprus” OR “czech republic” OR “denmark” OR “estonia” OR “france” OR “finland” OR “germany” OR “greece” OR “hong kong” OR “hungary” OR “iceland” OR “ireland” OR “israel” OR “italy” OR “japan” OR “kazakstan” OR “korea” OR “kuwait” OR “latvia” OR “liechtenstein” OR “lithuania” OR “luxembourg” OR “malta” OR “malaysia” OR “montenegro” OR “netherlands” OR “new zealand” OR “norway” OR “oman” OR “palau” OR “poland” OR “portugal” OR “qatar” OR “romania” OR “russia” OR “saudi arabia” OR “seychelles” OR “singapore” OR “slovakia” OR “slovenia” OR “spain” OR “sweden” OR “switzerland” OR “turkey” OR “united arab emirates” OR “united kingdom” OR “united states” OR “uruguay”

* used for word truncation.

## Appendix B

**Table A2:** Studies included in review.

Author	Year	Country	Setting/ Population	MCH Population	*n*	Primary or Secondary Analysis of Dataset Named (Year Conducted)	Study Design	FI Tool	Food Security Construct(s)
Alderete et al.	2018	Argentina	Food insecure participants of MCH programs in Primary Health Care clinics	Mothers of children < 1–6 years	*n* = 11	Primary (2015)	Qualitative	USDA HHFSM	Income and Employment Coping Strategies Food Access and Availability Other
Anderson et al.	2014	USA	Recently arrived Sudense refugees	Caregivers of children 0–3 years	*n* = 49	Primary (2002)	Cross-sectional	Radimer/Cornell Hunger Scale	Parent Acculturation Diet Quality
Cheu et al.	2020	USA	Women receiving prenatal care in a Chicago hospital	Postpartum women	*n* = 299	Primary (2018)	Cross-sectional	USDA HHFSM	Income and Employment Ethnicity Health Status Health Care
Chilton et al.	2009	USA	Immigrants and non-immigrants ED and pediatric care clinic users in 7 large cities	Mothers of children 0–3 years	*n* = 19,275	Children’s Sentinel Nutrition Assessment Program (1998–2005)	Cross-sectional	USDA HHFSM	Parent Acculturation
Cook et al.	2013	USA	Caregivers visiting primary/acute care clinics and EDs in 7 large cities	Caregivers of children 0–2 years	*n* = 41,515	Children’s Health Watch Survey (1998–2011)	Cross-sectional	USDA HHFSM	Family Composition Ethnicity Education Health Status Maternal Depression/Mental Health Parent Acculturation
Egeland et al.	2010	Canada	16 Nunavut Inuit communities	Caregivers of children 3–5 years	*n* = 388	Primary (2007–2008)	Cross-sectional	USDA HHFSM	Income and Employment Health Status Coping Strategies Residence Stability and Crowding
Findlay et al.	2013	Canada	Children identifying as Inuit	Caregivers of children 2–5 years	*n* = 1234	Aboriginal Children’s Survey (2006)	Cross-sectional	Single item	Education Coping Strategies Residence Stability and Crowding Smoking
Garg et al.	2015	USA	Nationally representative sample of children	Mothers of children at 9 & 24 months	*n* = 2917	Early Childhood Longitudinal Study, Birth Cohort (2001–2007)	Longitudinal	USDA HHFSM	Ethnicity Maternal Depression/Mental Health Participation in Food Assistance Programs
Hadley & Sellen	2006	USA	Recently arrived Liberian refugees	Caregivers of children 0–5 years	*n* = 33	Primary (unknown)	Cross-sectional	Radimer/Cornell Hunger Scale	Income and Employment Education Coping Strategies Parent Acculturation Participation in Food Assistance Programs Diet Quality
King	2017	USA	National sample of children, oversampling families in which mothers were unmarried at the birth of their child	Mothers of children at ages 3 & 5 years	*n* = 2481	Fragile Families and Child Wellbeing Study (1998–2000)	Longitudinal	USDA HHFSM	Income and Employment Ethnicity Coping Strategies Maternal Depression/Mental Health Participation in Food Assistance Programs
King	2018	USA	National sample of children, oversampling families in which mothers were unmarried at the birth of their child	Mothers of children at ages 3 & 5 years	*n* = 2044	Fragile Families and Child Wellbeing Study (1998–2000)	Longitudinal	USDA HHFSM	Family Composition Education Health Status Maternal Depression/Mental Health Smoking
Kreider et al.	2016	USA	National sample of adults and children in which vulnerable groups are oversampled	Mothers and children 0–5 years	*n* = 4614	National Health and Nutrition Examination Survey (1999–2008)	Cross-sectional	USDA HHFSM	Participation in Food Assistance Programs
Lindsay et al.	2009	USA	Immigrant, low-income Latina mothers	Mothers of children 0–48 months	*n* = 51	Primary (2005–2006)	Qualitative	None	Coping Strategies Maternal Depression/Mental Health Food Access and Availability
Lindsay et al.	2012	Argentina	Mothers with children attending child health and nutrition services	Mothers of children 2–5 years	*n* = 38	Primary (2006)	Qualitative	None	Coping Strategies Participation in Food Assistance Programs Food Access and Availability
Meyers et al.	2005	USA	Caregivers visiting pediatric clinics and EDs in 6 large cities	Caregivers of children 0–3 years	*n* = 11,723	Children’s Sentinel Nutrition Assessment Program (1998–2003)	Cross-sectional	USDA HHFSM	Health Status Residence Stability and Crowding
Morrissey et al.	2014	USA	Nationally representative sample of children	Caregivers of children at 9 months, 24 months, 4 years, and kindergarten entry	*n* = 11,700	Early Childhood Longitudinal Study, Birth Cohort (2001–2007) and data from the Council for Community and Economic Research (2001–2007)	Cross-sectional	USDA HHFSM	Food Access and Availability
Neault et al.	2007	USA	Immigrant mothers visiting pediatric clinics or EDs in 6 large cities	Mothers of children 0–12 months	*n*~3592 *n* = 3592	Children’s Sentinel Nutrition Assessment Program (1999–2004)	Cross-sectional	USDA HHFSM	Parent Acculturation Other
Park	2014	USA	National sample of adults and children in which vulnerable groups are oversampled	Pregnant women	*n* = 1045	National Health and Nutrition Examination Survey (1999–2010)	Cross-sectional	USDA HHFSM	Income and Employment Ethnicity Education Diet Quality Smoking Health Care
Patel & Surkan	2016	USA	Nationally representative sample of children	Caregivers of children at ages 9 & 24 months	*n* = 6150	Early Childhood Longitudinal Study, Birth Cohort (2001–2007)	Longitudinal	USDA HHFSM	Maternal Depression/Mental Health
Rosas et al.	2009	USA & Mexico	Mexican-born mothers and their children	Mothers of children 5 years	*n* = 602	Primary (2005–2006)	Cross-sectional	USDA HHFSM	Income and Employment Diet Quality
Schlichting	2019	New Zealand	Nationally representative sample of children	Caregivers of children 9 months	*n* = 6385 mothers, *n* = 6467 infants	Growing Up in New Zealand Study (2009–2010)	Cross-sectional (baseline data of a longitudinal survey)	New	Income and Employment Ethnicity Health Status Residence Stability and Crowding Diet Quality Smoking
Skalicky	2006	USA	Caregivers visiting a pediatric ED in Boston	Caregivers of children 0–3 years	*n* = 626	Children’s Sentinel Nutrition Assessment Program (1996–2001) & Iron Lab	Cross-sectional	USDA HHFSM	Income and Employment Education Health Status Residence Stability and Crowding Diet Quality
Tang	2020	USA	Latina mothers visiting a pediatric ED in Boston	Mothers of children 0–48 months	*n* = 2145	Children’s Health Watch Survey (2004–2013)	Cross-sectional	USDA HHFSM	Parent Acculturation
Trappmann	2015	USA	Caregivers of children attending Head Start in rural communities	Caregivers of children 3–5 years	*n* = 347	Primary (2008–2010)	Cross-sectional	Single item	Participation in Food Assistance Programs
Ward	2019	USA	Female caregivers of children attending Head Start	Female caregivers of children 3–5 years	*n* = 693	Primary (2006)	Cross-sectional	USDA HHFSM	Maternal Depression/Mental Health
Wong	2014	Malaysia	Mothers visiting 1 of 5 maternal and child health clinics in Terengganu	Mothers of children 0–5 years	*n* = 274	Primary (2012)	Cross-sectional	Radimer/Cornell Hunger Scale	Income and Employment Family Composition Education
Wong et al.	2019	Canada	Healthy urban children	Mothers of children 0–3 years	*n* = 3838	The Applied Research Group for Kids (TARGet Kids!) Longitudinal Cohort (2008–2016)	Cross-sectional	USDA HHFSM (2 item screener)	Income
Wu	2018	USA	Nationally representative sample of children	Caregivers of children at 9 months, 24 months, 4 years, and kindergarten entry	*n* = 6970	Early Childhood Longitudinal Study, Birth Cohort (2001–2006)	Cross-sectional	USDA HHFSM	Income and Employment Maternal Depression/Mental Health
Zalilah	2008	Malaysia	7 low-income, rural villages	Caregivers of children 1–6 years	*n* = 200	Primary (unknown)	Cross-sectional	Radimer/Cornell Hunger Scale	Income and Employment Family Composition Coping Strategies Residence Stability and Crowding
